# Clinical significance and integrative analysis of the cuproptosis-associated genes in head and neck squamous cell carcinoma

**DOI:** 10.18632/aging.204579

**Published:** 2023-03-20

**Authors:** Qiu Peng, Xianjie Jiang, Shiming Tan, Xuemeng Xu, Longzheng Xia, Nayiyuan Wu, Jinguan Lin, Linda Oyang, Yanyan Tang, Mingjing Peng, Min Su, Xia Luo, Yaqian Han, Qianjin Liao, Yujuan Zhou

**Affiliations:** 1 Hunan Key Laboratory of Cancer Metabolism, Hunan Cancer Hospital and The Affiliated Cancer Hospital of Xiangya School of Medicine, Central South University, Changsha 410013, Hunan, China; 2 Public Service Platform of Tumor Organoids Technology, Changsha 410013, Hunan, China

**Keywords:** HNSC, cuprotosis, cuprotosis-associated genes, cell death

## Abstract

Head and neck squamous cell carcinoma (HNSC) is a kind of malignant tumor originating from the oropharynx, larynx, nasopharynx and oral cavity. The incidence of HNSC is increasing and it is the sixth malignant tumor in the world at present. “Cuprotosis” is a novel cuper-dependent cell death mode that is closely related to mitochondrial respiration. Tumorigenesis is closely related to the dysregulation of cell death. However, the relationship between cuprotosis and HNSC remains unclear. Here, we investigated the association between 10 cuprotosis-associated genes (CAGs) and HNSC using multi-omics public data. We found that CAGs had abnormal expression and significant genetic changes in HNSC, especially CDKN2A with 54% mutation rate. Expression of CAGs significantly correlates with the prognosis of HNSC patients. Moreover, the CAGs expression is correlated with the immune checkpoints expression and immune cells infiltration. These CAGs expression was associated with multiple drugs sensitivity of cancer cells, such as cisplatin and docetaxel. These findings indicate that CAGs are likely to serve an essential role in the diagnosis, prognosis, immunotherapy and drug therapy prediction of HNSC.

## INTRODUCTION

HNSC originates in various parts of the head and neck, including the larynx, oral cavity, pharynx, and nasal cavity. There are more than 700,000 cases of HNSC every year worldwide [1]. It accounts for 5.3% of all cancers and is a serious threat to human health [2]. Smoking, drinking, poor oral hygiene and genetic factors are important risk factors for head and neck cancer. Studies have shown that the infection of HPV can also promote the development of HNSC, especially in oropharyngeal squamous cell carcinoma (OSCC) [3]. Although there are a large number of basic studies and treatment options for clinical selection, the 5-year mortality in patients with HNSC remains below 50% [4]. Recurrence or metastasis is the majority of reasons for the poor prognosis of patients after conventional chemotherapy, radiotherapy or combination therapy. [5]. Cisplatin-based chemotherapy is the primary treatment for HNSC patients with metastatic, but almost all such patients eventually suffer from treatment resistance or death within one year [6]. However, HNSC patients with metastatic is generally highly heterogeneous, and traditional chemotherapy has been unable to meet the needs of treatment, so it is urgent to explore a better treatment plan. Therefore, exploring more effective treatment methods is the key to improve the survival rate of HNSC patients.

Malignant tumor is a disease that seriously threatens human life and health. How to effectively kill tumor cells to increase the patient’s survival time is a difficult problem that the world has been trying to overcome. Cell death is usually considered to be of two categories: non-programmed death and programmed death [7]. Unprogrammed cell death refers to the uncontrolled cell death, also known as necrosis, which is usually the unexpected death of cells in response to physical or chemical stimuli in the environment and is characterized by cell swelling, plasma membrane rupture, and release of cellular contents [8]. Programmed cell death is a fine spontaneous death process controlled by genes, which can be divided into apoptosis, pyroptosis, autophagic death, iron death and necroptosis according to the morphological characteristics of the occurrence and the types of molecules involved [9–12]. Therefore, exploring different types of death modes will help to uncover specific regulatory molecules and provide new targets and treatment programs for tumor targeted therapy. It is expected to maximize the protection of normal cells while killing tumor cells for a precise treatment of cancer.

Recently, Tsvetkov et al. reported for the first time a novel copper-dependent cell death type “cuprotosis”, which was different from the apoptosis, necroptosis and pyroptosis, but similar to zinc-induced death and ferroptosis [13, 14]. Cuprotosis occurs when copper ions bind directly to the lipoacylated components of the tricarboxylic acid cycle during mitochondrial respiration, thereby promoting the clustering of lipoacylated proteins, followed by down-regulation of iron and sulfur clusters, and finally leading to proteotoxic stress to promote cell death [13]. The occurrence of cuprotosis is regulated by pro-cuproptosis genes (DLD, DLAT, FDX1, LIAS, LIPT1, PDHA1 and PDHB) and anti-cuproptosis genes (CDKN2A, GLS and MTF1) [15]. Further research on which cells are more susceptible to cuprotosis may help to develop new treatments for cancer.

Metal ion-induced death, such as zinc and iron [16, 17], is closely associated with tumorigenesis and progression, but the relationship between cuproptosis and head and neck tumors is unclear. Here, we analyzed the CAGs genetic changes and expression in head and neck tumors using public databases. We proved the relevance of the expression of CAGs to the prognosis, infiltration of immune cells and multidrug sensitization in HNSC. These results suggest that members from CAGs may serve as promising prognostic and therapeutic biomarkers of HNSC.

## MATERIALS AND METHODS

### CAGs expression profile analysis

The RNAseq data of HNSC were acquired from TCGA database. We further analyzed the differential expression of CAGs in 504 tumor samples and 44 normal samples using the R software to analyse the downloaded data [18].

### CAGs genetic alteration analysis

Genetic mutation data for HNSC were obtained by downloading from the cbioportal database [19, 20]. Analysis of the somatic mutations in HNSC was performed in R software. Genes with higher mutational frequency detected of HNSC patient in histogram was showed.

### Correlation analysis between CAGs expression and HNSC patient’s prognosis

Sequencing data and patient information related to HNSC were extracted from the TCGA database, from which data in TPM format were extracted, and then normalized log2 (TPM+1) was performed, and finally samples with RNAseq data and clinical information were retained. Finally, 504 HNSC samples were used for subsequent analysis. KM survival analysis was examined using log ranks to compare survival between the two or more groups described above, and a time ROC analysis was performed to define the accuracy of the discriminant prediction model [21–24].

Lasso: Lasso regression algorithm was applied to characteristic selection and 10-fold cross-validation was used. The above analysis was carried out using R software glmnet package.

Cox: The prognostic model was constructed using multifactorial Cox regression analysis, and the analysis was carried out using the R software survival package.

Step: The optimal model was selected as the final model through multi-factor Cox regression analysis and then iterative analysis by Step function.

### Predictive nomogram of CAGs in HNSC

Univariate and multivariate analyses allow the identification of variables that can be used as Nomogram plots. If the gene is associated with a significant difference in prognosis in both univariate and multivariate, it can be indicated as a variable independent of other clinical factors. The closer the Nomogram model is to the calibration curve indicates that the model predicts better results.

### Correlation analysis of CAGs and immune cell infiltration

Sequencing data and patient information related to HNSC were extracted from the TCGA database. R software was utilized for multi-gene correlation testing [25–29]. CTLA4, TIGIT, LAG3, SIGLEC15, PDCD1, CD274, HAVCR2 and PDCD1LG2 are associated with immune checkpoint. The correlation between CAGs and the expression values of these 8 genes was analyzed by R software. Potential immunotherapy response is anticipated by the TIDE algorithm [30, 31].

### Correlation analysis of CAGs and drug sensitivity

Based on the largest publicly available GDSC pharmacogenomic database (https://www.cancerrxgene.org/), chemotherapy response was determined using the R package to make predictions for each HNSC sample [32–34].

### Immunohistochemical analysis

Cuproptosis-related genes were entered in the Human Protein Atlas database [35] and used the immunohistochemical results therein to analyze the protein level expression of cuproptosis-related genes in head and neck tumor samples and normal samples.

### Availability of data and materials

TCGA publicly available datasets were analyzed in this study.

## RESULTS

### Somatic alteration and abnormal expression of multiple CAGs in HNSC

**Figure 1:**
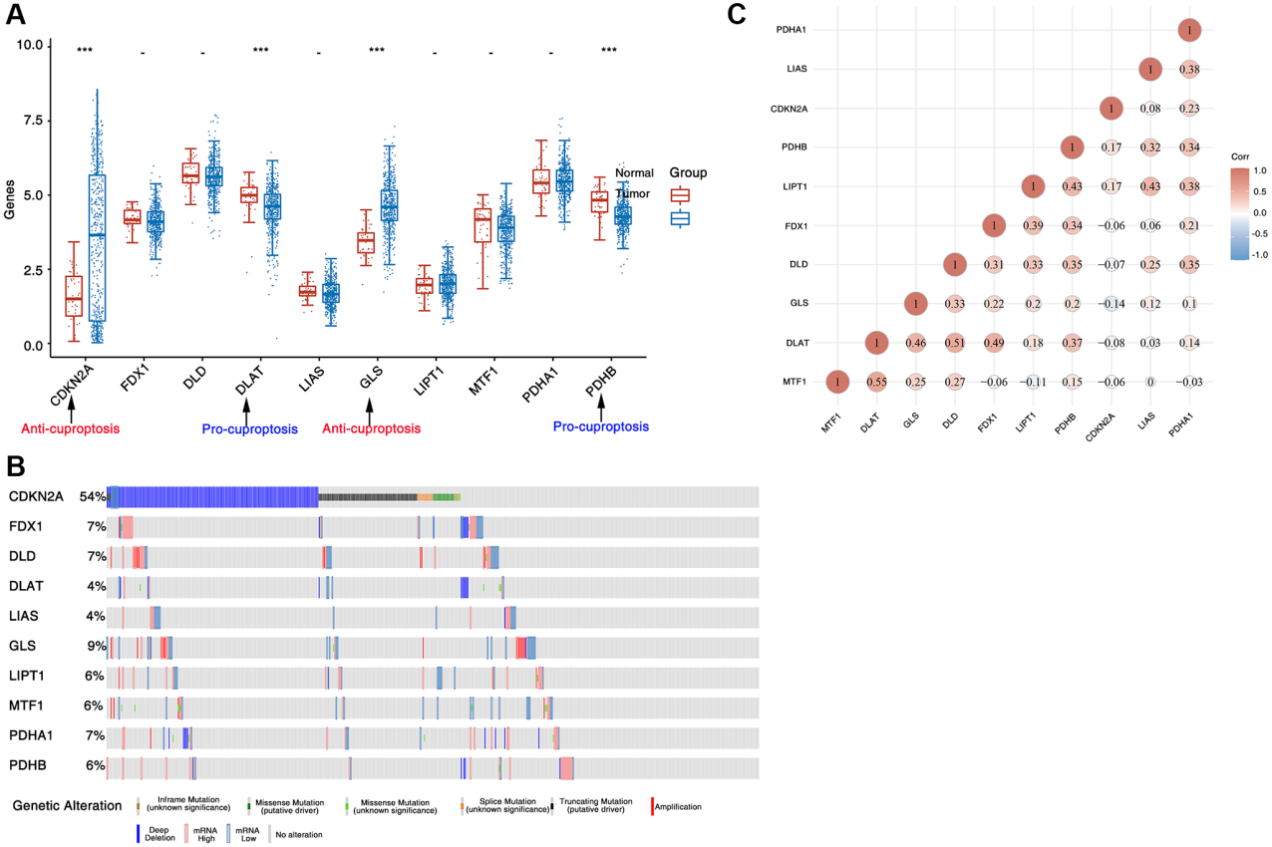
**Expression and genetic alteration of CAGs in HNSC.** (**A**) The CAGs expression in HNSC tissues and normal tissues. (**B**) The mutation frequency of CAGs in HNSC. The horizontal coordinates indicate the different sample groups and the vertical coordinates indicate the ratio of CAGs mutations. (**C**) Correlation of CAGs in HNSC. ^*^stands for significance levels, ^*^for *p* < 0.05.

**Figure 2:**
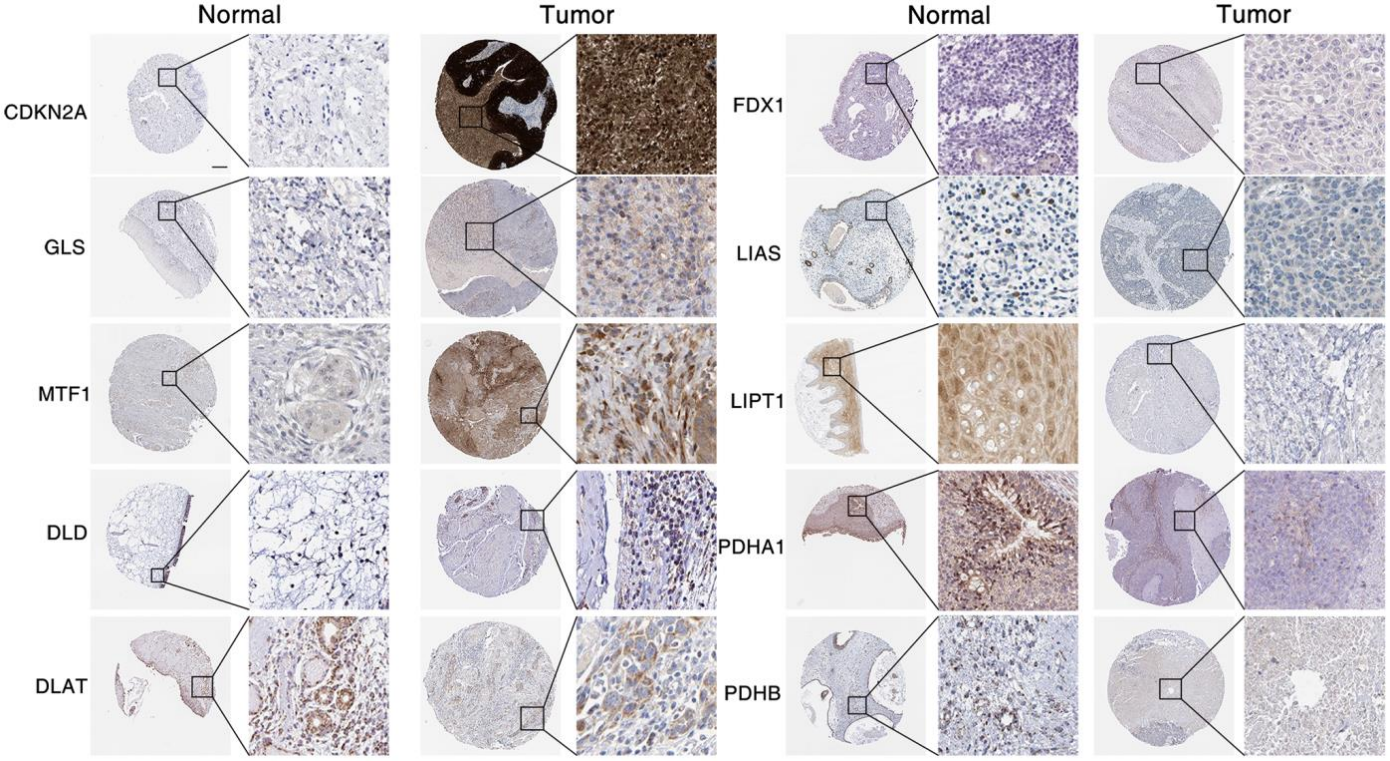
**Expression of CAGs in HNSC.** CAGs expression data from The Human Protein Atlas database in tumor with or normal tissue were measured by immunohistochemical staining. Scale bar, 20 μm.

To evaluate the 10 CAGs expression levels of seven pro-cuproptosis genes (LIPT1, DLD, FDX1, PDHA1, DLAT, LIAS and PDHB) and three anti-cuproptosis genes (CDKN2A, GLS and MTF1) in head and neck tumor tissues and normal tissues, we used TCGA data analysis and found that the anti-cuproptosis genes CDKN2A and GLS in tumor tissues were remarkably highly elevated in expression levels. In contrast, the expression levels of the pro-cuproptosis genes DLAT and PDHB were obviously reduced in tumor tissues as compared to normal tissues (Figure 1A). Similarly, using clinical samples from The Human Protein Atlas database, we also found that pro-cuproptosis genes tended to be under-expressed in tumors, while anti-cuproptosis genes were generally over-expressed (Figure 2). To detect the genetic changes of CAGs, we downloaded HNSC data from cBioPortal database, and found that there were obvious mutations in these genes (the lowest mutation frequency was 4% of DLAT and LIAS), especially in CDKN2A, whose mutation frequency was up to 54% (Figure 1B). The main mutation forms include deep deletion, missense mutation and amplification. We also examined correlations between cuprotosis-associated genes and found significant correlations between these genes (Figure 1C). These results show CAGs are abnormally expressed and have significant genetic changes in HNSC, indicating that CAGs are likely to be important in HNSC.

### CAGs predict the prognosis of HNSC patients

**Figure 3:**
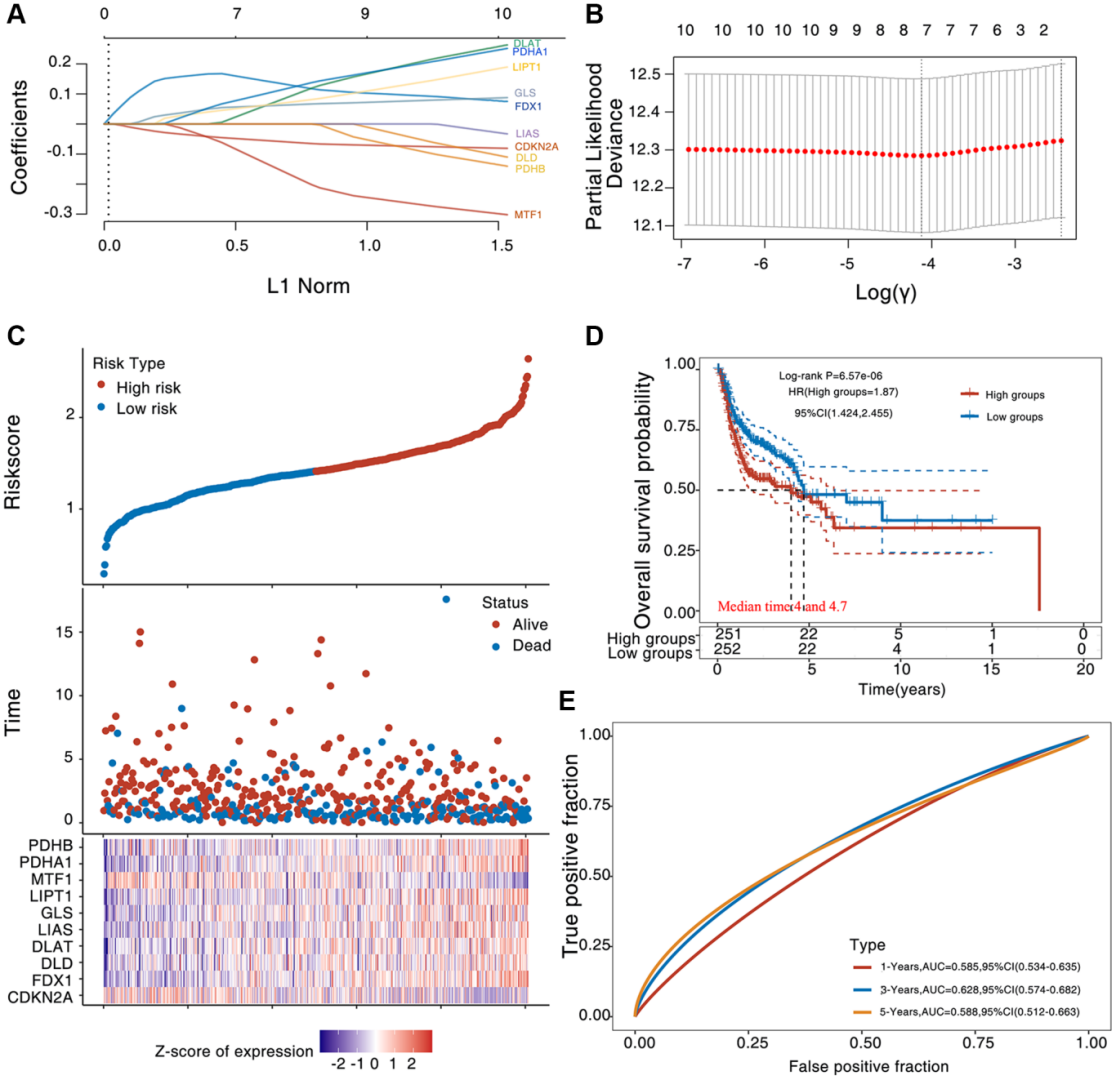
**Prognostic of CAGs in HNSC.** (**A**) The factors of the elected characteristic are shown as lambda parameters. (**B**) The partial likelihood deviance was plotted versus log (λ) using the LASSO Cox regression model. (**C**) Riskscore, survival time and survival status in the selected data set. (**D**, **E**) The KM survival curve distribution of the risk model in the data set, among which different groups were tested by log rank (**D**). The ROC curve and AUC of the risk model at different times (**E**), where the higher the AUC value, the stronger the predictive ability of the model.

**Figure 4:**
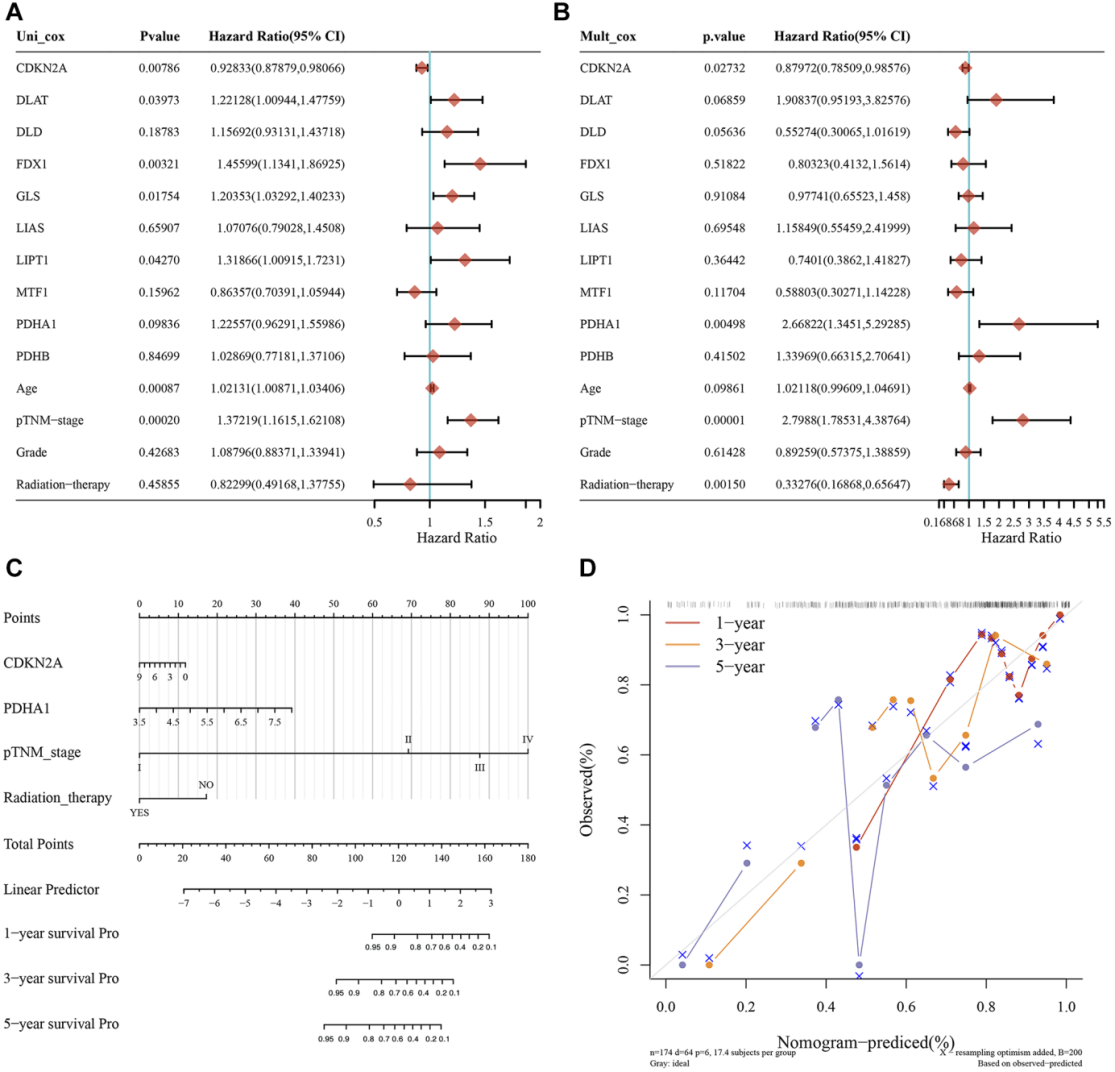
**Predictive nomogram of CAGs in HNSC.** (**A**, **B**) Univariate (**A**) and multivariate (**B**) COX analysis were used to determine the *P*-value, HR, and confidence intervals of 10 CAGs expression and clinical characteristics. (**C**) The 1-, 3-, and 5-year overall survival rates of HNSC patients were predicted by Nomogram. (**D**) The diagonal dashed lines represent the ideal nomogram.

To assess the relevance of CAGs expression to HNSC prognosis, a dimensionality reduction based on Lasso or multivariate Cox iterative regression was performed and a prognostic model was constructed. CAGs were defined as prognostic risk elements (Figure 3A, 3B). We have grouped HNSC patients according to CAG expression, HNSC survival status and risk score into high-risk and low-risk groups (Figure 3C). By Kaplan-Meier survival assay, we found that the overall survival of patients in the high-risk group was significantly lower than that of patients in the low-risk group (Figure 3D). We found that the AUC scores for 1, 3 and 5 years were 0.589, 0.65 and 0.623, respectively by analyzing ROC curves at different times (Figure 3E). Moreover, we also structured a predictive nomogram with CAGs and clinical characteristics (including age, pTNM stage, grade, radiation therapy of HNSC patients). By univariate and multivariate cox regression analysis, an independent prognostic factor for HNSC patients was found to be the expression levels of CDKN2A and PDHA1, pTNM staging and radiotherapy (Figure 4A, 4B). The nomogram prediction model showed that overall survival at 3 and 5 years could be predicted relatively well in the whole cohort compared with the ideal model (Figure 4C, 4D). These results show that CAGs may be used as prognostic biomarkers in HNSC patients.

### CAGs expression is related with immune checkpoint, immune cell infiltration and immune checkpoint blockade (ICB) response in HNSC

**Figure 5:**
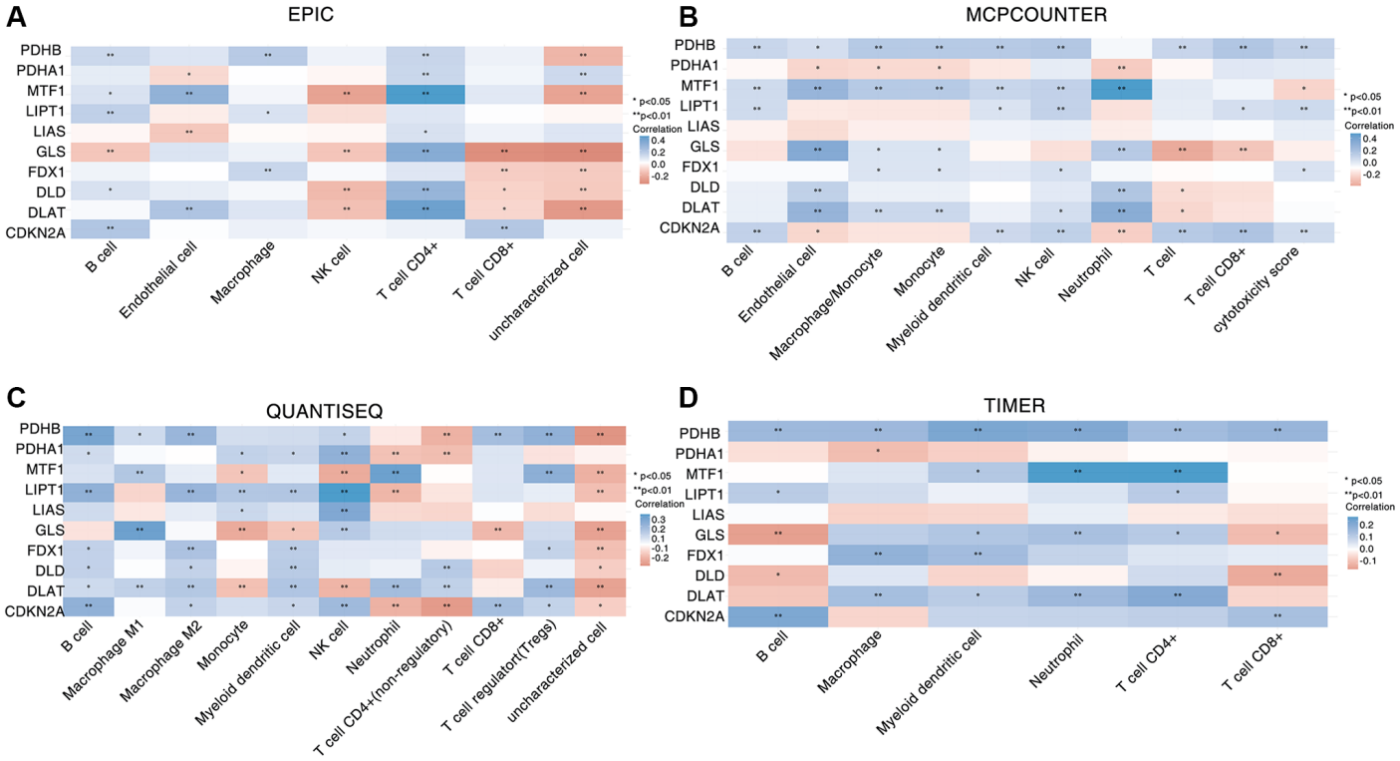
**Correlation between CAGs expression and various immune cells infiltration of HNSC.** (**A**–**D**) Four algorithms including EPIC (**A**), MCP-counter (**B**), quanTIseq (**C**) and TIMER (**D**) to evaluate the correlation between CAGs and immune cell infiltration. The ordinate represents CAGs, the abscissa represents different immune cells. ^*^stands for significance levels, ^*^for *p* < 0.05.

To detect the connection for CAGs and tumor immunity, we analyzed the relationship to the CAG expression and immune cell infiltration using TIMER, McP-counter, EPIC and quanTIseq immune scoring algorithms. We found that most CAGs were involved in infiltrating B cells, T cells, NK cells and macrophages (Figure 5A–5D).

**Figure 6:**
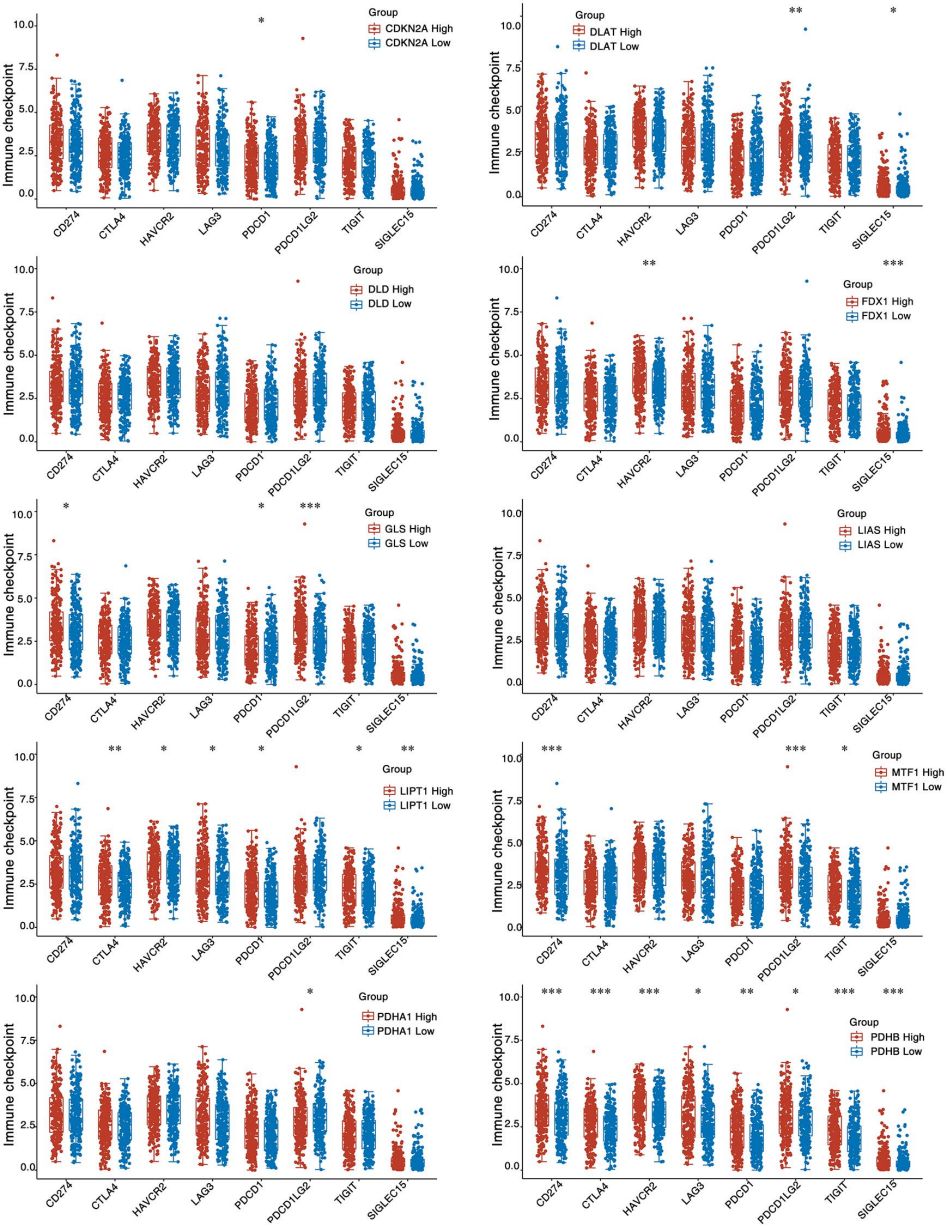
**Expression distribution of immune checkpoint of HNSC samples with differential CAGs expression.**^ *^stands for significance levels, ^*^for *p* < 0.05.

Immune checkpoint molecules play a suppressive role in the immediate immune system and are critically important for preventing autoimmune responses, sustaining self-tolerance, and minimal damage to organs. Immune cell function can be inhibited by immune checkpoint molecules, preventing cells from producing a potent defense against tumor immunity and creating an immune escapement of the tumor [36]. To detect the correspondence from CAGs to immune checkpoints, we divided the HNSC samples into high and low CAGs expression categories, and analyzed the level of expression of common immune checkpoint molecules including CD274, TIGIT, PDCD1LG2, HAVCR2, PDCD1, CTLA4, LAG3 and SIGLEC15 in these two groups. We found that PDHB and LIPT1 were correlated with almost all of the eight immune checkpoints. CDKN2A, DLAT, FDX1, GLS, MTF1 and PDHA1 were also associated with more than one immune checkpoint (Figure 6).

**Figure 7:**
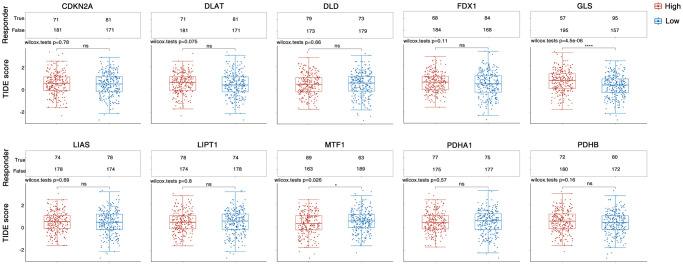
**TIDE algorithm predicted the immune checkpoint inhibitor response of HNSC samples with differentially expressing CAGs.** The upper panel represents the statistical tables of immune responses of samples. Lower panel indicates the distribution of immune response scores. ^*^stands for significance levels, ^*^for *p* < 0.05.

In recent years, ICB has radically changed the way cancer is treated in humans. To explore the correlation between CAGs and ICB response, we project the response of HNSC samples differentially expressing CAG to predicted immune checkpoint inhibitors using the TIDE algorithm [31]. HNSC patients with differential GLS and MTF1 expression had a significant correlation with ICB response (Figure 7). These measurements imply that CAGs potentially serve an invaluable role in the immunotherapy of HNSC patients.

### Expression of CAGs is correlated with drug sensitivity

**Figure 8:**
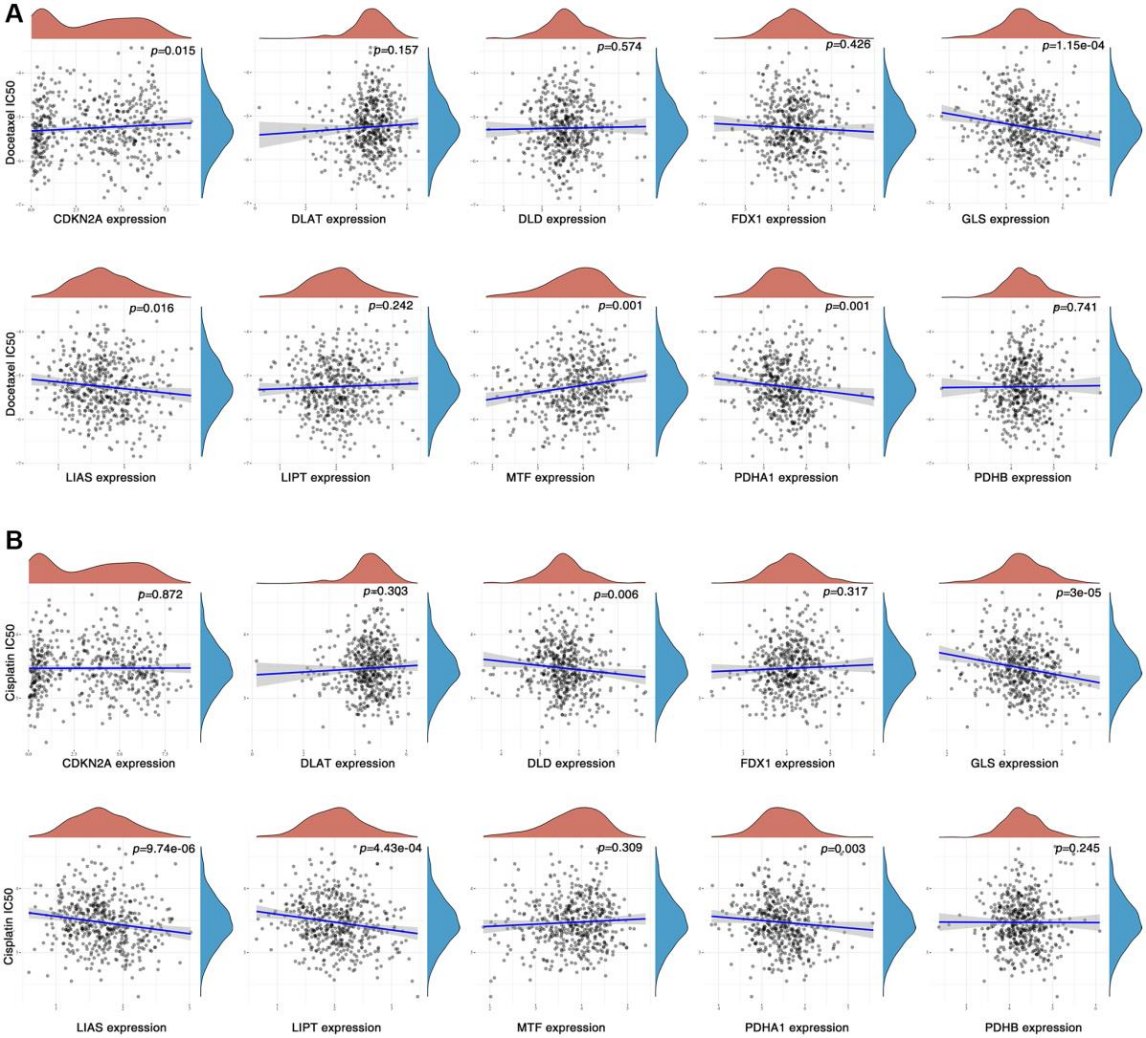
**Correlation between CAGs expression and drug sensitivity.** (**A**, **B**) Spearman correlation analysis of cisplatin (**A**) docetaxel (**B**) IC50 score and CAG expression.

IC50 (half inhibitory concentration) is an important index for evaluating drug efficacy or sample response to treatment. To test the association of CAGs with common drugs currently used to treat HNSC patients, including cisplatin and docetaxel, we used the GDSC database, the biggest public pharmacogenomics database, based on the transcriptome data of HNSC sample and predicted the drug response of CAGs in differentially expressed HNSC patients. We found that the expressions of CDKN2A, GLS, LIAS, MTF and PDHA1 were dramatically correlated for cisplatin sensitivity in HNSC patients (Figure 8A). Expressions of DLD, GLS, LIAS, LIPT and PDHA1 were dramatically correlated for the docetaxel sensitivity in HNSC patients (Figure 8B). These data implied that CAGs could be an effective factor in the drug sensitivity of HNSC patients.

## DISCUSSION

Head and neck cancer is a malignant tumor originating from different organs of the head and neck, such as tongue, oral cavity, pharynx, paranasal sinuses, accounting for about 4% of primary tumors in the whole body. Head and neck malignancies are mainly squamous cell carcinoma, which is currently ranked as the 6th most common cancer worldwide [1]. Studies have reported that the primary danger elements for HNSC are smoking and alcohol consumption [37, 38]. In recent years, despite the continuous improvement of surgery, radiotherapy, chemotherapy and other comprehensive treatment techniques, there are still many patients with distant metastasis within 5 years [39]. Therefore, it is a matter of major relevance to study the prognostic elements of HNSC and their mechanisms.

Recently, studies have reported that copper dependent controlled cell death (cuproptosis) is a novel cell death mode different from apoptosis, pyroptosis, ferroptosis and other cell death modes. Cuprotosis occurs when copper ions bind directly to the lipoacylated components of the lipoacylated tricarboxylic acid cycle during mitochondrial respiration, thereby promoting the aggregation of lipoacylated proteins, followed by down-regulation of iron and sulfur clusters, and finally leading to proteotoxic stress to promote cell death [15]. The occurrence of cuprotosis is regulated by many genes, including mainly pro-cuproptosis genes such as DLD, DLAT, FDX1, LIAS, LIPT1, PDHA1 and PDHB, and anti-cuproptosis genes such as CDKN2A, GLS and MTF1. Recent studies have found that these CAGs may be associated with the development of diverse tumors, including melanoma, renal cell carcinoma, glioma, hepatocellular carcinoma, soft tissue sarcoma, lung cancer, colorectal cancer and so on [40–48]. Other findings have also identified that cuproptosis-associated lncRNA may predict the prognosis of HNSC [49, 50], but whether CAG can predict the prognosis, immune cell infiltration and drug sensitivity of HNSC has not been reported. Here, we investigated the CAGs expression and genetic changes in HNSC using public databases. We confirmed the interaction of CAGs expression with prognosis, infiltration of immune cells and multidrug sensitivity in HNSC. Further research on which cells are more susceptible to cuprotosis may help to develop new treatments for HNSC.

Cisplatin-based chemotherapy is the primary treatment for patients with recurrent or metastatic HNSC, but almost all such patients eventually suffer from treatment resistance or death within one year [6]. Traditional chemotherapy has been unable to meet the needs of treatment, so it is urgent to explore a better treatment plan. Here, we found that CAGs were significantly correlated with cisplatin and docetaxel sensitivity by database prediction. Therefore, whether the development of drugs targeting CAG gene can increase the sensitivity of chemotherapy drugs in HNSC patients is a meaningful research direction.

Immunotherapy provides a new approach for head and neck tumors, including tumor vaccines, cytokines, immune cell activation and immune checkpoint modulators. Among them, immune checkpoint inhibitors have achieved significant survival benefits, programmed death protein-1 inhibitors pembrolizumab and opdivo are approved for second-line treatment of recurrent HNSC [51, 52]. In our work, we revealed the expression of CAGs was strongly correlated with the infiltration of immune cells, the expression of immune checkpoint molecules, and the ICB response. This suggests that the development of drugs targeting CAGs and combined immunotherapy may be more effective in the treatment of HNSC patients.
